# Supplementary material for: Clinical viability of magnetic bead implants in muscle
Source: Front Bioeng Biotechnol. 2022 Oct 25;10:1010276. doi: 10.3389/fbioe.2022.1010276 (PMC9640959; doi:10.3389/fbioe.2022.1010276)
Supplement: Supplementary file 1 [file DataSheet1.PDF]

Supplementary Material

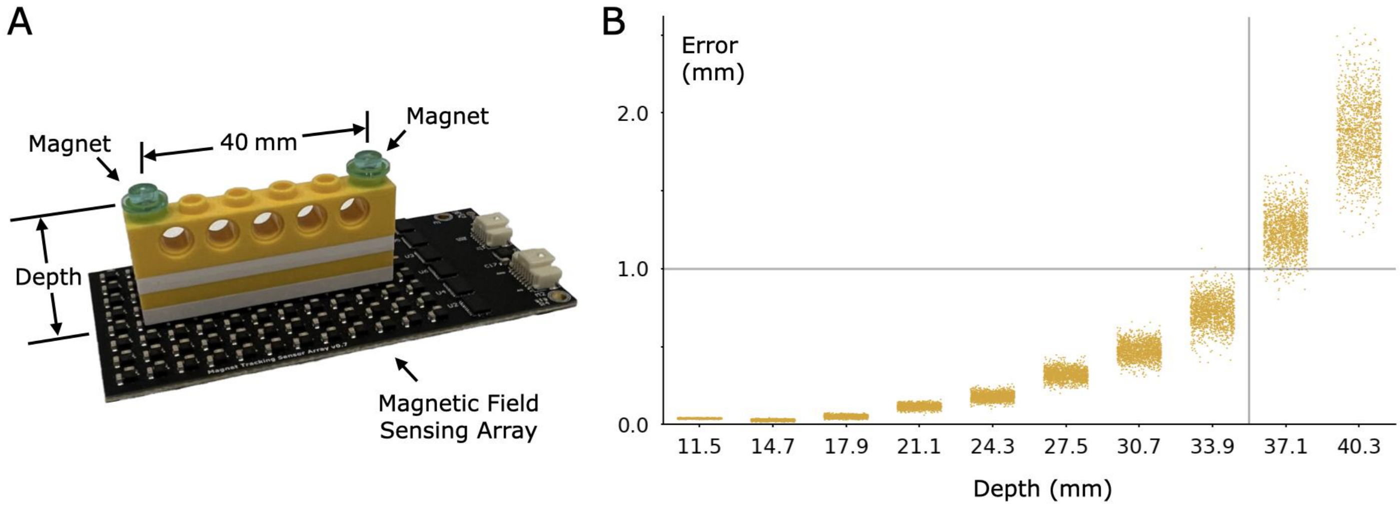

Supplementary Figure 1: Magnetomicrometry Sensing Error vs Implantation Depth of Two Implantable Magnetic Beads. ***(A)*** *We affixed two magnetic beads (manufactured as described in the methods) into 1x1 LEGO round plates, and we attached these two round plates 40 mm apart to a 1x6 LEGO technic brick. We then centered this LEGO brick over a 96-element (LIS3MDL) magnetic field sensing array parallel with the long axis of the array. We varied the depth in 3.2 mm increments from the initial depth of 11.5 mm by adding 1x6 LEGO plates. This centering presents a best-case scenario, suggesting maximum depth limits that could be achieved with proper placement of magnetic field sensors.* ***(B)*** *Seaborn strip plots show the error in mm (vertical axis) of the magnetomicrometry signal for each depth. The horizontal axis shows each depth categorically. Note that the magnetomicrometry best-case accuracy with this sensing array and these magnets is sub-millimeter to a depth of about 33 mm.*

Supplementary Table 1: Initial and Final Separation Distances Between Implanted Magnets. *The following table lists the various magnetic bead separation distances sorted in ascending order by initial separation distance. We implanted magnetic bead pairs into the lateral gastrocnemius (LG) and tibialis cranialis (TC) of the right (R) legs of turkeys D-G. We implanted a magnetic bead pair into the right LG and three magnetic beads into the left (L) LG of turkey H. For the left LG of turkey H, we have listed all three pairwise initial and final separation distances between the three implanted magnetic beads. We implanted magnetic bead pairs into the femoralis tibialis (FT) and the LG of both legs of turkey I and in the left leg of turkey J.*

Separation (mm)		
**Initial**	**Final**	**ID/Leg/Muscle**
15.7	2.8	I/L/LG
16.6	3.5	J/L/LG
18.3	16.6	J/L/FT
20.1	3.4	I/R/FT
21.2	24.5	I/L/FT
23.4	21.7	I/R/LG
29.0	28.2	H/L/LG (2 to 3)
36.9	36.9	G/R/TC
37.2	36.8	H/L/LG (1 to 2)
38.2	41.4	D/R/LG
38.4	40.8	F/R/TC
43.5	45.5	G/R/LG
44.7	45.4	F/R/LG
47.5	58.7	H/R/LG
51.9	60.8	D/R/TC
65.3	64.3	H/L/LG (1 to 3)
66.6	69.0	E/R/LG
70.2	71.8	E/R/TC
